# Immediate Rehabilitation of Critical-Size Gunshot- and Mine Blast-Related Maxillary Defects Using Cortically Anchored Single-Piece Implants: Two Case Reports

**DOI:** 10.3390/reports9030257

**Published:** 2026-08-06

**Authors:** Yan Vares, Yarema Vares, Łukasz Pałka, Raphael Olszewski

**Affiliations:** 1 Department of Maxillofacial Surgery, The Danylo Halytsky Lviv National Medical University, 69 Pekarska Str., 79010 Lviv, Ukraine; vares-dent@ukr.net (Y.V.); raphael.olszewski@saintluc.uclouvain.be (R.O.); 2 Superhumans Center, 31 Ivasiuk Str., 79495 Lviv-Vynnyky, Ukraine; varesyarema@gmail.com; 3 Department of Operative Surgery and Topographic Anatomy, The Danylo Halytsky Lviv National Medical University, 69 Pekarska Str., 79010 Lviv, Ukraine; 4 Private Dental Practice, Rzeszowska 2, 68-200 Żary, Poland; 5 Department of Oral and Maxillofacial Surgery, Cliniques Universitaires Saint-Luc, UCLouvain, 1348 Brussels, Belgium; 6 Oral and Maxillofacial Surgery Research Lab (OMFS Lab), NMSK, IREC, UCLouvain, 1348 Brussels, Belgium; 7 Department of Perioperative Dentistry, Ludwika Rydygiera Collegium Medicum in Bydgoszcz, Nicolaus Copernicus University in Torun, 85-067 Torun, Poland

**Keywords:** case report, maxillofacial trauma, gunshot injury, mine blast injury, alveolar defect, cortical anchorage, immediate loading

## Abstract

**Background and Clinical Significance:** Implant rehabilitation of patients with acquired maxillofacial defects remains challenging, particularly following high-energy war-related trauma. Gunshot and mine blast injuries frequently result in extensive hard and soft tissue loss, often requiring complex reconstructive procedures. Although cortically anchored implants have been successfully used in patients with severe maxillary atrophy and selected traumatic defects, evidence supporting their use for the immediate rehabilitation of critical-size war-related maxillary defects remains limited. Cortically anchored single-piece implants used in conjunction with an immediate loading protocol may provide an alternative rehabilitation strategy for selected patients who decline, or are unsuitable for, conventional implants and bone-grafting procedures. **Case Presentation:** Two patients with critical-size maxillary defects (approximately 3 cm) resulting from gunshot and mine blast injuries are presented. Treatment consisted of extraction of non-restorable teeth, placement of cortically anchored single-piece implants, including tubero-pterygoid implants, followed by immediate loading with fixed hybrid metal–acrylic hybrid prostheses. Clinical and radiological evaluation was performed using panoramic radiography and cone-beam computed tomography. **Conclusions:** Successful implant-supported prosthetic rehabilitation was achieved in both patients. Cortically anchored implants engaging the basal bone of the maxilla provided stable support for immediately loaded fixed prostheses despite substantial hard and soft tissue loss. Functional and aesthetic outcomes were satisfactory. Immediate prosthetic rehabilitation was successfully completed in both patients. A 12-month clinical and radiographic follow-up was available for one patient and demonstrated stable implant function without biological or prosthetic complications. Long-term follow-up of the second patient was not available because of active military service. Cortically anchored implant-supported hybrid prostheses may represent a viable treatment option for selected patients with critical-size maxillary defects resulting from gunshot or mine blast injuries, enabling rapid restoration of oral function and facial aesthetics while avoiding extensive bone-grafting procedures.

## 1. Introduction and Clinical Significance

Since the onset of the military conflict in Ukraine in February 2022, healthcare providers have been required to manage an unprecedented number of military and civilian patients with gunshot- and mine blast-related maxillofacial injuries [1,2]. These injuries frequently result in extensive hard and soft tissue destruction of the craniofacial region, often accompanied by tooth loss, leading to functional impairment, facial disfigurement, psychological distress, and a substantial reduction in quality of life [3]. Depending on their size and location, alveolar and maxillary defects often require reconstruction using autogenous, allogeneic, xenogeneic, or synthetic grafting materials [4]. Critical-size jaw defects (generally exceeding 3 cm) are commonly managed using vascularized autogenous tissue grafts. Although these procedures represent the current standard of care, they are associated with donor-site morbidity, additional surgical trauma, prolonged postoperative recovery, and extended rehabilitation periods.) Following successful graft integration, dental implants can be placed in the reconstructed bone and restored according to conventional delayed loading protocols, typically after 3–6 months [5]. In wartime conditions, where the number of patients requiring complex maxillofacial reconstruction may increase rapidly, treatment strategies that reduce the number of surgical procedures and shorten rehabilitation time are essential for preserving healthcare resources and facilitating earlier functional recovery.

The increasing use of cortically anchored implant systems with an immediate loading protocol has expanded the therapeutic options for patients with severe alveolar atrophy, post-oncological defects, traumatic maxillofacial injuries, and jaw bone reconstruction [6,7,8,9,10].

Although favorable outcomes have been reported with cortically anchored implants in patients with jaw defects after trauma, evidence regarding their use for the immediate rehabilitation of critical-size war-related maxillary defects remains limited.

Therefore, the aim of this report is to describe the rehabilitation of two patients with critical-size maxillary defects resulting from gunshot and mine blast injuries using cortically anchored single-piece implants, including tubero-pterygoid implants, followed by immediate loading with fixed hybrid prostheses.

## 2. Case Presentation

The present cases are reported in accordance with the Surgical CAse REport (SCARE) 2025 guidelines [11].

### 2.1. Case 1

A 54-year-old male military serviceman was admitted on 17 March 2025 for implant-supported prosthetic rehabilitation and revision of intraoral scar tissue. According to the patient’s medical records, he sustained a mine blast injury near Bakhmut (Donetsk region, Ukraine) on 27 December 2022. The injury resulted in traumatic brain injury with concussion (without central nervous system dysfunction), a shrapnel wound to the right side of the face, a comminuted fracture of the maxilla associated with an approximately 2.5 cm hard palate defect, and multiple fractured and missing teeth. First aid was provided on the battlefield. On 28 December 2022, surgical treatment of the craniofacial injuries was performed, including removal of a retained foreign body from the left temporal region. On 24 May 2023, surgical closure of the oronasal fistula was performed.

**Figure 1:**
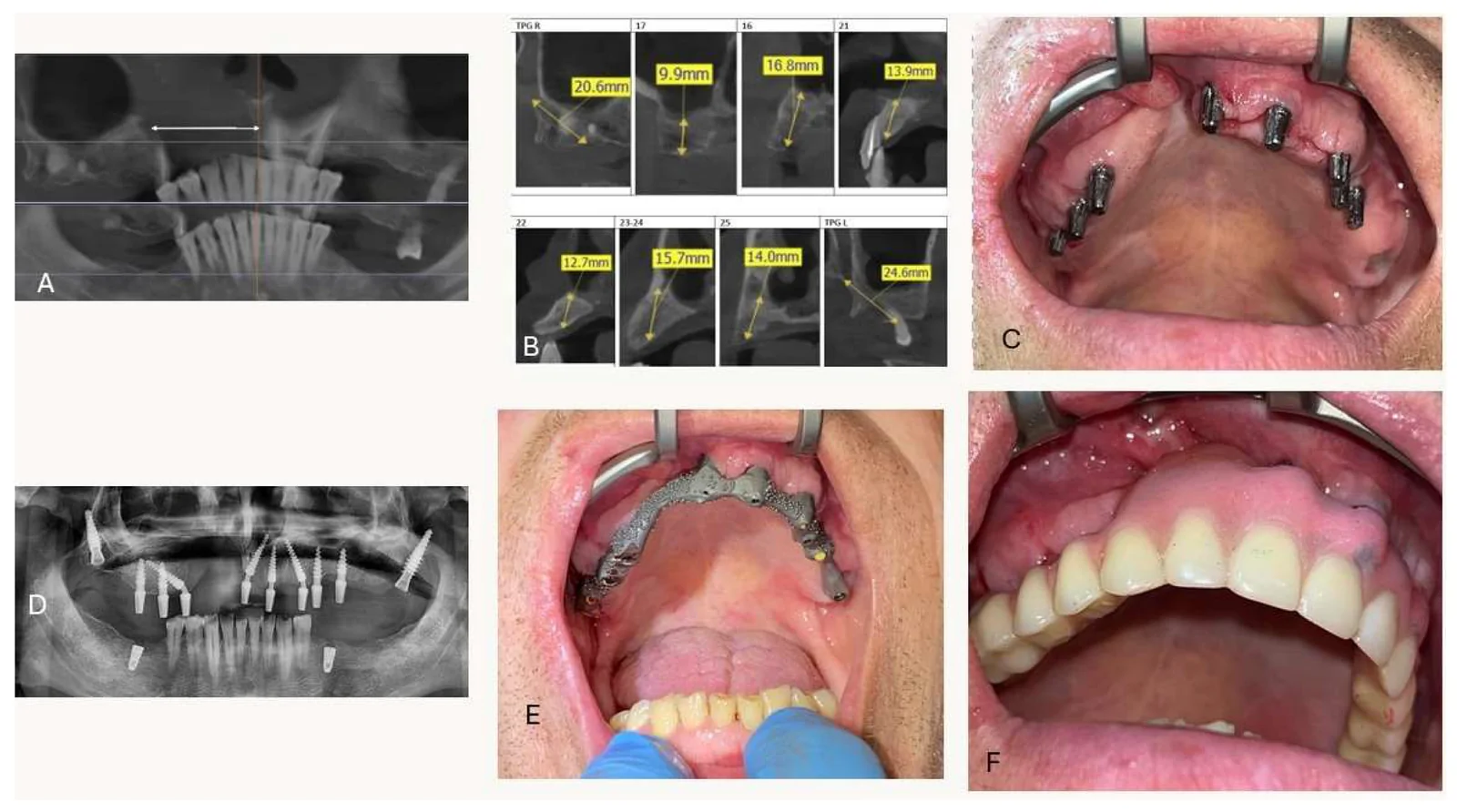
Clinical and radiographic management of Case 1. (**A**) Pre-operative CBCT screen with visualization of the maxillary defect. (**B**) Measurements of the height of the alveolar process in potential implant sites. (**C**) Intraoral view after removal of 18, 14, 21, 23, and 28 teeth, bone reduction in maxillary frontal area and placement of 10 implants. (**D**) Post-operative panoramic X-ray with visualization of implant location. (**E**) Intraoral view. Framework try-in. (**F**) View of the hybrid prosthesis in the mouth.

On 18 March 2025, a CBCT scan (Vatech Pax-i3D Green, Vatech Co., Ltd., Hwaseong, Gyeonggi, Republic of Korea, 94 kV, 7,7 mA, slice thickness 0.18 mm, field of view 16.9 cm) of the facial bones was performed. The CBCT images were analyzed using EZ3D software Version 5.4 (Vatech, Republic of Korea). Measurements of the maxillary defect and available bone height at the planned implant sites, particularly in the tuberosity and pterygoid regions (pterygomaxillary junction) (Figure 1A,B) were performed by a single examiner (Y.Y.V.).

On 19 March 2025, under general anesthesia, the patient underwent extraction of the retained roots of teeth 18, 14, 21, 23, and 28, followed by alveolar ridge reduction in the region of teeth 21–23. Subsequently, eight cortically anchored single-piece compression implants (KOS^®^ X and KOS^®^ Micro, Ihde Dental AG, Switzerland) and two tubero-pterygoid implants (TPG^®^, Ihde Dental AG, Switzerland) were placed (Figure 1C).

Most compression screw implants were placed bicortically, engaging the cortical plates of the nasal floor of the left maxillary sinus, whereas the tubero-pterygoid implants achieved anchorage in the cortical bone of the pterygoid processes of the sphenoid bone. The surgical sites were closed using 4-0 Monocryl sutures.

During the same surgical session, impressions were obtained using transfer caps and Speedex C-Silicone putty (Coltene, Switzerland). Amoxicillin/clavulanate, paracetamol, and dexamethasone were administered perioperatively. Postoperative care included analgesic therapy and antiseptic mouth rinses. Implant positioning was verified by postoperative panoramic radiography (Figure 1D).

On 24 March 2025, a try-in of the cobalt–chromium framework was performed (Figure 1E). Two days later, on 26 March 2025, the sutures were removed and the definitive metal–acrylic hybrid prosthesis was delivered under an immediate loading protocol (Figure 1F).

At the latest follow-up examination (twelve months after prosthesis delivery), the patient remained free of implant mobility issues or biological or prosthetic complications. Clinical examination demonstrated healthy peri-implant soft tissues and satisfactory functional and aesthetic outcomes, while radiographic evaluation confirmed stable implant positioning without evidence of peri-implant radiolucency.

### 2.2. Case 2

**Figure 2:**
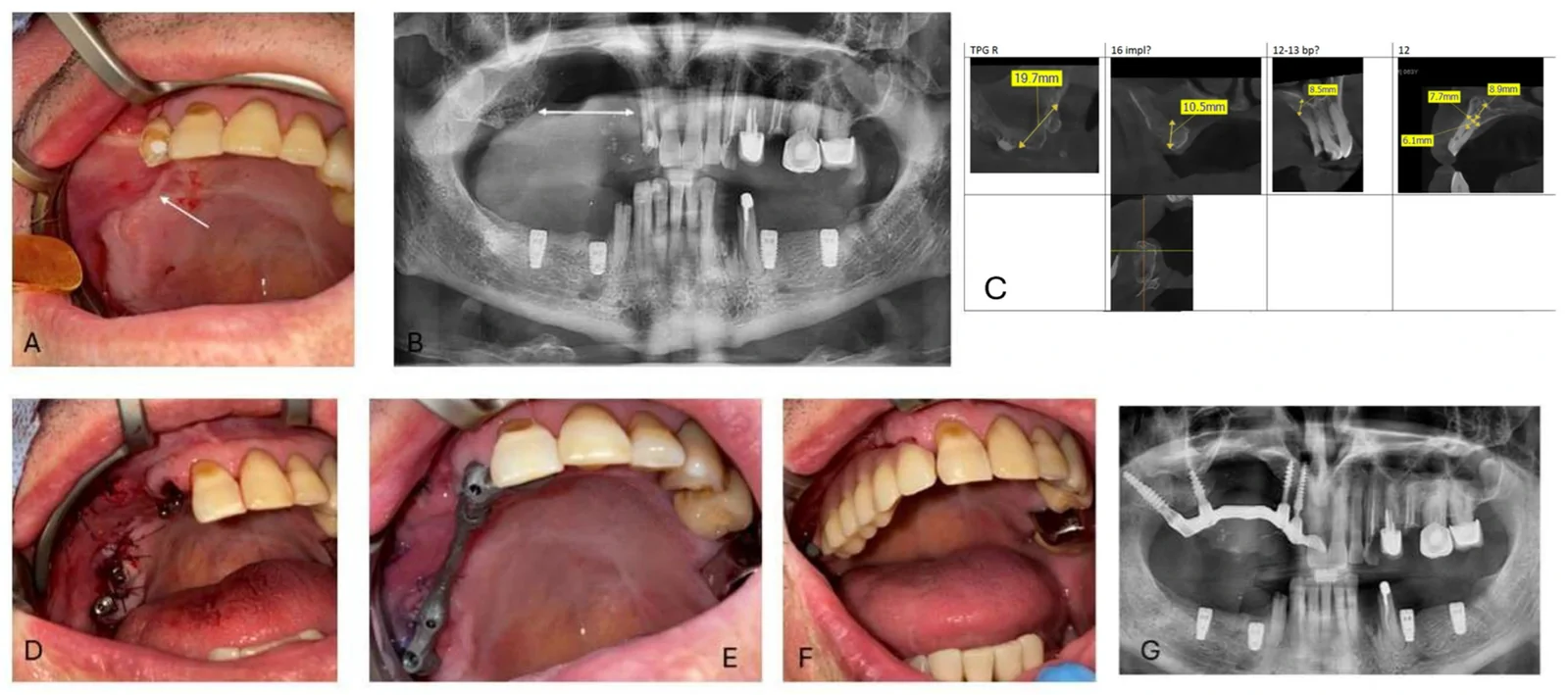
Clinical and radiographic management of Case 2. (**A**) Visualization of maxillary defect on the right side. (**B**) Panoramic X-ray on admission day. Defect of the maxilla on the right side. (**C**) Measurements of the height of the alveolar ridge of the upper jaw at the sites of potential implant placement. (**D**) Intraoral view after removal of tooth 12, placement of 4 single-piece implants and vestibuloplasty on the right side. (**E**) Intraoral view. Framework try-in. (**F**) View of the hybrid prosthesis in the mouth. (**G**) Post-operative panoramic X-ray with visualization of implant location and framework fitting.

A 64-year-old male military serviceman was admitted on 15 August 2025 for implant-supported prosthetic rehabilitation. The admission diagnosis was a gunshot-induced fracture of the right maxilla with a hard tissue defect and partial secondary tooth loss affecting both the maxillary and mandibular arches (Figure 2A,B).

According to the patient’s medical records, he sustained a gunshot shrapnel injury to the face while carrying out a combat mission near Klishchiivka (Donetsk region, Ukraine) on 18 November 2023. The injury resulted in multiple fractures of the right facial skeleton associated with extensive soft tissue loss, a gunshot injury to the right upper extremity requiring traumatic amputation at the elbow, abdominal penetrating trauma, traumatic brain injury, and concussion. On the day of the injury, the patient underwent emergency surgical management of the gunshot wounds, exploratory abdominal surgery, and amputation of the right upper limb. On 29 November 2023, surgical closure of the oroantral fistula and reconstruction of the right oral commissure were performed.

On 16 August 2025, cone-beam computed tomography (CBCT) of the facial skeleton was performed using a Vatech Pax-i3D Green unit (Vatech, Republic of Korea; 94 kV, 7.7 mA, slice thickness 0.18 mm, field of view 16 × 9 cm). The CBCT images were analyzed using EZ3D software (Vatech, Republic of Korea). Measurements of the maxillary sinus defect and the available bone height at the planned implant sites, particularly in the maxillary tuberosity and pterygoid regions (pterygomaxillary junction), were obtained to assess the feasibility of implant placement (Figure 2C).

On 21 August 2025, under local anesthesia, tooth 12 was extracted. Four cortically anchored single-piece implants (KOS, KOS Micro, BCS, TPG (Ihde Dental AG, Switzerland) were then placed in the region of the missing teeth 12, 13, 17, and 18, bypassing the osseous defect. Simultaneously, vestibuloplasty of the right vestibule was performed (Figure 2D). During the same surgical session, impressions were obtained using C-Silicone (Speedex, Coltene, Switzerland) for fabrication of a metal–acrylic hybrid prosthesis.

On 26 August 2025, a try-in of the cobalt–chromium framework was performed (Figure 2E). Two days later, on 28 August 2025, the sutures were removed and the definitive metal–acrylic hybrid prosthesis was delivered under an immediate loading protocol (Figure 2F). Postoperative panoramic radiography confirmed satisfactory positioning of all four implants and accurate adaptation of the metal–acrylic hybrid prosthesis (Figure 2G).

## 3. Discussion

Rehabilitation of patients with gunshot or mine blast injuries of the maxillofacial region requires a multidisciplinary approach involving trauma surgeons, maxillofacial surgeons, prosthodontists, and rehabilitation specialists [4,12,13]. Contemporary treatment protocols generally consist of staged debridement, reconstruction of hard and soft tissue defects using local flaps or vascularized autogenous grafts, followed by delayed prosthetic rehabilitation after adequate healing [13]. Although these approaches remain the standard of care, restoration of oral function with implant-supported prostheses continues to present significant clinical challenges [14,15]. There are well-known and well-documented cases of short and ultra-short implants with a delayed loading protocol being placed in fibula or iliac bone grafts in the presence of large defects of the upper or lower jaw [5,16]. Although vascularized fibular and iliac crest free flaps have traditionally been used to restore skeletal continuity in extensive maxillary defects, recent reports highlight zygomatic implants as a predictable, graftless alternative for achieving stable dental rehabilitation [17]. The main disadvantages of such operations are a significant mismatch between the crestal level of the native jawbone and the graft, which is currently partially solved by the double fibula free flap technique (double barrel fibula flap) [18], as well as the presence of a thick layer of mobile mucosa over the integrated implants, which requires its excision and the use of free gingival grafts [19]. The negative aspect of this type of treatment is the multi-stage nature of the procedures and the need for long waiting periods associated with implant integration, which is not always possible for patients who are active military personnel. These limitations highlight the need for alternative treatment strategies capable of reducing the number of surgical procedures while providing rapid functional rehabilitation.

The concept of cortically anchored single-piece implants with immediate loading has demonstrated favorable clinical outcomes in patients with advanced periodontal disease [20], severe alveolar ridge atrophy [6,7], and selected systemic conditions [21,22].

Successful rehabilitation using cortically anchored implants has also been reported in patients with subtotal post-oncological defects of the maxilla and mandible [8,9,10]. Furthermore, Awadalkreem et al. described successful immediate rehabilitation of severe maxillofacial trauma caused by road traffic accidents using corticobasal implant-supported fixed prostheses without bone grafting, reporting stable long-term function and excellent patient satisfaction [23].

Reports describing the use of cortico-basal implants for ballistic facial injuries remain scarce. We identified one published report describing the long-term results of the functioning of six cortico-basal screw implants in a patient with a significant post-traumatic defect of the lower jaw [24]. The authors note the excellent condition of the peri-implant tissues, the reliable stability of the prosthetic construction, and the high level of patient satisfaction. More recently, Duman et al. described successful staged rehabilitation of a mandibular gunshot injury using vestibuloplasty followed by delayed implant-supported prosthetic rehabilitation after multiple reconstructive procedures [25]. In contrast to this previously published report, which described rehabilitation of a single patient with a post-traumatic mandibular defect, the present report extends the existing evidence by illustrating the feasibility of immediate rehabilitation of critical-size maxillary defects resulting from contemporary war-related injuries using cortically anchored single-piece implants, including tubero-pterygoid anchorage.

Implant placement in the presence of severe bone deficiency, extensive vertical defects, or compromised bone quality should be performed in accordance with established cortico-basal implantology protocols [26,27]. In the present cases, particular emphasis was placed on achieving stable distal cortical anchorage using long tubero-pterygoid implants, with their apical portions engaging the cortical bone of the pterygoid processes of the sphenoid bone. The biomechanical advantages of distal cortical anchorage using pterygoid implants have recently been confirmed in a systematic review demonstrating high survival rates and predictable outcomes in rehabilitation of severely compromised posterior maxillae without bone grafting [28]. Single-piece smooth-surface implants and rough- surface compression implants were placed bicortically, engaging the cortical plates of the nasal floor and the maxillary sinus whenever anatomically feasible [29]. Immediate splinting of the implants with a rigid metal framework (within 3–5 days after surgery) likely contributed to favorable biomechanical load distribution and enhanced prosthetic stability during functional loading. The acrylic component of the hybrid prosthesis facilitated rapid restoration of facial aesthetics by compensating for the existing tissue defect and providing support for the surrounding soft tissues.

A limitation of the present report is the lack of uniform long-term follow-up. While one patient was evaluated clinically and radiographically 12 months after treatment, follow-up of the second patient was not possible because he remained on active military duty. Such challenges are common in conflict-related medical care and should be considered when interpreting the long-term outcomes.

## 4. Conclusions

Cortico-basal implant-supported hybrid prostheses are a feasible treatment modality for the rehabilitation of patients with critical-size gunshot or mine blast defects of the maxillofacial area, which allow for the rapid restoration of the aesthetic and functional status of the face and enable patients, particularly military personnel, to return to their professional duties. This approach may represent a valuable alternative rehabilitation strategy for carefully selected patients in whom extensive grafting procedures are contraindicated, declined by the patient, or impractical because of the clinical circumstances. Further studies with larger sample sizes and follow-up periods are recommended.

## Data Availability

The data presented in this study are available from the corresponding author upon reasonable request.
